# Urban housing prices, female labor participation, and economic development in china: A theoretical and empirical analysis

**DOI:** 10.3389/fpsyg.2022.970039

**Published:** 2023-01-04

**Authors:** Yuan Li, Jamal Khan, Qaiser Jamal Mahsud

**Affiliations:** ^1^Institute of International Studies, Shandong University, Weihai, Shandong, China; ^2^School of Northeast Asia Studies, Shandong University, Weihai, Shandong, China; ^3^Department of Public Administration, Hazara University, Mansehra, Pakistan

**Keywords:** urban housing prices, female labor participation, economic development, threshold effect, China

## Abstract

China's housing distribution system has undergone a major transformation, and the country's housing markets have experienced a rapid price increase. However, the extent to which urban housing prices influence female labor participation (FLP) in labor decision-making and how the FLP rate affects economic development has not been sufficiently investigated. Accordingly, we first build a theoretical neoclassical economic development model that includes housing consumption factors to estimate the effect of housing price dynamics on FLP. We then use the 2017 China Family Panel Studies (CFPS) database to empirically estimate the intrinsic relationship between urban housing prices, FLP, and economic development through the lens of the balanced growth path, and we come up with four main findings. First, the theoretical model demonstrates that rising housing prices increase FLP, stimulating economic development. However, an excessive increase in housing prices will undermine women's ability to drive economic development. Second, the empirical evidence shows that a unit increase in housing prices increases the probability of FLP by 0.186%. Third, the effects of housing prices on economic development vary across China's Eastern, Central, and Northeastern regions. Finally, the threshold model shows that FLP positively influences economic development until the housing price logarithm reaches 8.8134, after which FLP's beneficial effect on economic development will be diminished.

## Introduction

China's double-digit phenomenal economic growth, which has been fueled by China's opening up and reform, has been accompanied by substantial structural changes in the country's housing and labor markets. The total amount and structure of labor participation in the labor market have shifted dramatically, and women's participation in economic activity, in particular, has undergone significant changes as a result of their labor market participation (Fu et al., 2016). The country had one of the highest female labor force participation (FLP) rates of 73.2% in Asia-Pacific in the 1990s^1^, which even though has steadily declined to 60.5% in 2019. By comparison, the male labor force participation rate increased from 61 to 75.3% (World Bank, 2022). From the aspect of international comparison, China's labor participation rate was 76% in 2018, ranking first in the world. Chinese FLP rates between the ages of 25 and 55 were extremely high, particularly in urban China, with working married women having a 5–18% higher participation rate than unemployed married women (Chen et al., 2018).

Along with rapid economic growth and significant labor market changes, the Chinese housing market has undergone significant changes, and land-centered development is a key feature of its regional development (Zhu et al., 2019). Housing reforms in the 1990s, combined with the triple process of widespread rural–urban migration, accelerated urbanization, and rapid industrial growth, resulted in a massive increase in housing demand, resulting in a significant rise in housing prices (Duarte et al., 2019). For example, average housing prices increased from 1,948 to 3,576 RMB per square meter in 2000 and 2008 to 7,614 RMB per square meter in 2017 (Li et al., 2018). However, rising housing prices have an impact on China's labor market, particularly FLP, because a higher relative cost of housing encourages households to increase labor supply by bringing two earners into the labor market (Warren and Tyagi, 2003). As a result of the higher labor inputs, increased FLP becomes a key driver of economic development, and the economy may be able to grow more quickly as more women enter the workforce. At the same time, FLP increases household incomes, allowing families to escape poverty and increase their spending on goods and services (Duarte et al., 2019).

Given the dual nature of housing consumption and investment, as well as the heterogeneous impact of housing prices on the labor market (particularly on the FLP), both of which affect economic development. Hence, determining the extent to which urban housing prices affect FLP in labor decision-making and how the FLP rate affects economic development is policy relevant. This would allow policymakers to gain a better understanding of the social and economic consequences of housing price dynamics in China, allowing them to design effective policies to optimize labor market and housing market decisions while also ensuring long-term economic growth.

We pursue three objectives in this study. Our first objective is to figure out how housing price dynamics affect FLP in China. Second, we aim to assess the inner linkage between FLP and economic development. Third, we aim to quantify the non-linear relationship between FLP and economic development in order to establish a housing price ceiling and mitigate the negative effects of rising housing prices on economic development.

The empirical question of whether household wealth has an effect on labor market behavior is critical in economics. Housing wealth accounts for a sizable portion of household wealth, prompting numerous studies on the relationship between labor supply and housing wealth (Jiang et al., 2021). Numerous researchers believe that rising housing cost encourages households to increase labor supply by bringing two earners into the labor market (Warren and Tyagi, 2003; Li, 2019; Zhao, 2019). In this context, the question of what effect urban housing prices have on FLP rates has received considerable attention from researchers (see Section 2 for the review of relevant literature). To answer this question, scholars focus on both the theoretical mechanism that the housing price affects the FLP rate (Jiang et al., 2021) and the logical exploration of experience to understand how urban housing prices affect the FLP rates (Fu et al., 2016; Adelino et al., 2017; Jansson, 2017).

The majority of studies in this diverse literature have separately examined the dynamic effects of housing prices on FLP, as well as the relationship between FLP and economic growth. In addition, no unified theoretical framework exists for the logical relationship between housing prices, FLP, and economic development. More particularly, the internal connection and dynamic mechanism between housing prices and FLP are rarely examined from a fundamental theory perspective. Furthermore, previous studies used GDP (or per capita GDP) and the quadratic term of GDP (or per capita GDP) as variables when analyzing the inverted U-shaped relationship between FLP rate and economic development, without examining whether there are multiple threshold effects. This study, therefore, attempts to deduce the internal relationship between housing prices and FLP and explain the logical and more comprehensive relationship between housing prices, FLP, and economic development.

We use the equilibrium path analysis method to develop an economic development model and examine how FLP affects economic growth *via* the housing price variable in the process of utility maximization under budget constraints. Subsequently, we employ the threshold effect model and other measurement techniques to examine the mechanism by which FLP affects the national economy, allowing us to provide some guidance for future policy formulation. We use data from the 2017 China Family Panel Studies (CFPSs) database (micro-database) for the empirical analysis. CFPS data are notable in that it tracks and collects data samples from 31 provinces and autonomous regions in China, excluding Hong Kong, Macao, and Taiwan, to reflect changes in China's society, economy, population, education, and health.

Our theoretical and empirical analysis yields the following main results. First, the theoretical model shows that an increase in housing prices leads to an increase in FLP, which stimulates economic growth. However, FLP and economic development have an inverted U-shaped relationship. Second, according to the empirical analysis, every one-unit increase in housing prices increases the likelihood of FLP by 0.186%. Third, at the regional level, housing prices have a positive heterogeneous impact on economic development in Eastern, Central, and Northeastern China. Finally, the threshold model demonstrates that the economic development of FLP can be positively influenced until the house price logarithm reaches 8.8134. After 8.8134, FLP's beneficial effect on economic development efficiency will be harmed.

We make 3-fold contributions to the existing body of research. First, we develop an economic development model using equilibrium path analysis and investigate how FLP affects economic growth *via* the housing price variable in the process of utility maximization under budget constraints. Second, we make use of the newest 2017 CFPS dataset, which includes detailed information on each household's housing and other assets, as well as demographic data. These comprehensive statistics enable us to quantify the ability of each household to supply female labor. Finally, we examine a non-linear relationship between FLP and economic development using Hansen (1999) threshold effect model. It will enable us to provide policy guidance on how to reach an agreement on a housing price cap, thereby mitigating the economic development-depressing effects of rising housing prices.

**Figure 1:**
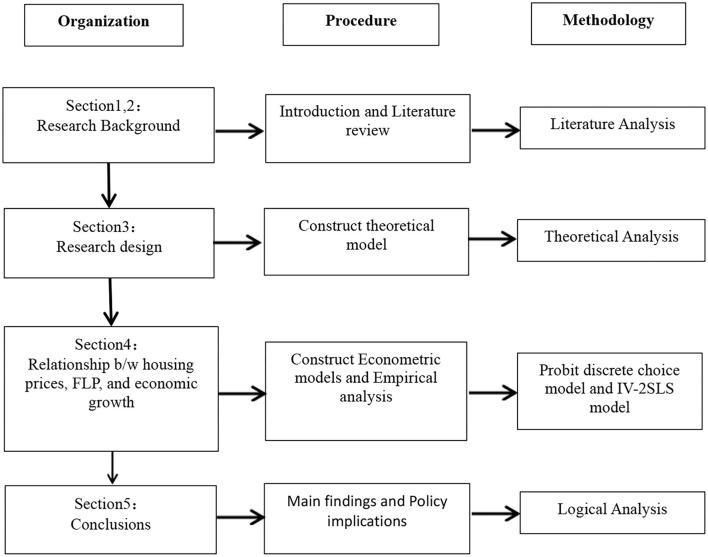
Organization of the study.

The remainder of the study is organized in the following manner (see Figure 1). Section 2 presents a review of the literature. Section 3 presents the theoretical framework and sheds light on the data. Section 4 contains the empirical analysis and major findings, and Section 5 concludes.

## Literature review

Housing is both a consumer and an investment good, accounting for a sizable portion of most households' wealth. Policymakers attempting to influence the labor market and academics attempting to understand household decision-making have paid close attention to the impact of changes in housing wealth due to price fluctuations on household labor supply (Doorn et al., 2019; Luo and Yao, 2021). Some academics argued that the relationship between urban housing costs and labor supply is bidirectional (Johnson, 2014), with higher relative housing costs encouraging households to send two earners into the workforce and rising two-earner families driving up land prices, which in turn raise relative housing costs (Gyourko et al., 2010, 2013; Zhang and Zhang, 2017). The rising housing costs have “push-pull” effects on labor decisions. On the one hand, rising housing costs have a crowding-out effect on labor mobility. For example, Ganong and Shoag (2017) discovered that rising housing costs in a location (such as cities) discourage the migration of low-skilled workers. At the same time, others found that higher housing costs in a region prevent labor migration inflow (Plantinga et al., 2013) and push laborers to migrate to other regions with lower housing costs (Guerrieri et al., 2013). On the other hand, rising housing costs may have a clustering effect on the labor force. Higher housing costs in cities attract highly skilled workers, who will have more employment opportunities, earn higher wages, and receive greater benefits (Zhou et al., 2020). Moreover, the anticipated wealth effect of rising housing prices encourages workers to migrate to cities (Zhao and Li, 2015; Li et al., 2021).

In recent decades, rising FLP and housing prices have been the two most noticeable changes in particular, and labor economists have centered a considerable amount of their research efforts on these two issues. It is argued that higher housing prices are the major factor of rising FLP. For example, Warren and Tyagi (2003) argued in their popular book “The Two Income Trap” that rising housing prices induce households to supply more labor to the market by sending two earners into the labor market, thereby increasing FLP (Tripathi, 2019).

Several empirical studies have been carried out to determine the relationship between housing prices and FLP, with mixed results (Henley, 2004; Farnham and Sevak, 2007; Disney and Gathergood, 2009). In the United Kingdom, for example, Henley (2004) discovered that rising housing prices result in a decline in FLP. Likewise, Disney and Gathergood (2009) demonstrated that rising housing prices have a negative effect on the FLP and work hours of young homeowners in the United Kingdom. Moreover, Milosch (2014) demonstrated that rising housing prices reduce married FLP, with the effect being greater for married women with a high income and education. In comparison, other studies have demonstrated that rising housing prices result in an increase in living costs, which increases FLP (Johnson, 2014; He, 2015). Using data from the British Household Panel Survey, He (2015) found that rising housing prices lead to an increase in the country's FLP. However, other studies found no evidence of the relationship between housing prices and FLP. For example, Black et al. (2014) examined the variation in married women's labor force participation across 50 metropolitan areas and discovered that a portion of the variation is attributable to commuting costs. They find no correlation between rising housing prices and FLP. Likewise, Johnson (2014) concluded that housing prices in the United States have a minimal or negligible impact on FLP.

In China, numerous theoretical and empirical studies on the impact of urban housing on FLP have been conducted (Wu et al., 2014, 2016; Chen and Tran, 2018; Fan et al., 2019; Liu et al., 2019; Jiang et al., 2021). For example, Wu et al. (2014) investigated the effect of housing prices on FLP using CHNS data. Their findings reveal that a 1% increase in China's house prices would result in a 0.08% decline in overall FLP. The net effect of prices on an FLP varied according to age, marital status, schooling years, and whether the family had a baby. Li and Wu (2014) discovered that while high housing prices increase wealth, they discourage urban adults from starting their own businesses. In addition, Fu et al. (2016) estimated the effect of housing price dynamics on FLP in China. They concluded that a rise of 10,000 RMB in housing value reduces the likelihood of female homeowners joining the labor force by 1.37 percentage points, while increasing the likelihood of becoming housewives by 1.49 percentage points. Similarly, Zhao et al. (2018) discovered that rising housing prices boost the labor supply in urban China. They also concluded that rising housing prices have a greater impact on women and the younger labor supply. In addition, Jiang et al. (2021) recently developed a theoretical model to make inquiries about the impacts on labor supply stemming from housing wealth in the context of the Chinese housing boom. They concluded that rising housing prices reduce working hours—most notably for women—but have no effect on employment.

It may seem intuitive to assert that the dynamics of housing prices affect FLP, which in turn affects economic growth. A large body of evidence suggests that FLP and economic growth have a U-shaped relationship (Tam, 2011; Lechman and Okonowicz, 2013; Tsani et al., 2013; Verme, 2015; Ma et al., 2017). Both are conditioned by country structural transformations (Boserup et al., 2013). On the one hand, the declining portion of the U-shaped curve explains the transition from subsistence agriculture to industrialized and labor-intensive economies, where more male input is required than female input. On the other hand, the increasing portion of the U-shaped curve represents the process of increasing FLP, which is typical as the national economy becomes more service-based (Gaddis and Klasen, 2012). Based on a comprehensive sample of data from 130 countries, Tam (2011) concluded that FLP in various industries had a heterogeneous economic development impact and that FLP could promote per capita GDP growth at a certain stage but inhibit it once a certain threshold was crossed. Tsani et al. (2013) reached a similar conclusion.

## Theoretical model

### Basic model

It is assumed that an economy is composed of an (urban) family, which consists of two individuals, a male and a female, labeled m and f (i = m, f), with similar preferences and the same initial endowment. Suppose they are married with children in the family (not limited to how many children they have). For taking care of the children, there are irreplaceable parts that must be taken by the mother, and parts can be replaced by the father or mother. The time that must be taken by the mother is denoted by *t*_0_. Meanwhile, assuming that *a*_*i*_ represents the consumption of the individual *i* and *c* is the initial endowment. The utility function of the individual can be expressed as follows:


(1) $$\begin{array}{l} U_{i}=U_{i}\left(a_{i},c,t\right) \end{array}$$

where *U*_*i*_ represents the utility obtained by individual *i* from consumption, and *t* represents time. The individual's human capital is *h*_*i*_. The quality of human capital improves with experience acquisition, and the default is that there is no gender difference. The rate of increase in human capital is υ per hour. Assuming that, individual “*i*” works for “*L*” hour, his salary can be expressed as follows:


(2) $$\begin{array}{l} w_{i}=\left(1+\upsilon L_{i}\right)h_{i}\omega \end{array}$$

where ω represents the wage rate of individual *i*. There is no difference of ω between male and female individuals as a return of human capital. There exists a tradeoff between family care and work, while there is zero leisure time. In order to facilitate the analysis, each individual's working time is unitized, that is, the total working time is 1. The working hours of female and male individuals are as follows:


(3) $$\begin{array}{l} L_{f}=1-t_{0}-t_{f} \end{array}$$

(4) $$\begin{array}{l} L_{m}=1-t_{m} \end{array}$$

where *t*_*f*_ and *t*_*m*_ represent the time spent by women and men per unit of time in the family, respectively, such as caring for children, assuming that *t*_*f*_ and *t*_*m*_ can be replaced entirely:


(5) $$\begin{array}{l} t_{f}+t_{m}=t \end{array}$$

Here, we consider that “*t*” will not be massive; otherwise, women will not have enough time to take care of their children (this is the irreplaceable part), then:


(6) $$\begin{array}{l} t+t_{0}\leq 1 \end{array}$$

The composition of consumption, it can be known that the consumption budgets of representative women and men can be expressed as follows:


(7) $$\begin{array}{l} B_{f}=\theta \left(P_{R}R+M\right) \end{array}$$

(8) $$\begin{array}{l} B_{m}=\left(1\text{-}\theta \right)\left(P_{R}R+M\right) \end{array}$$

where consumption expenditure of female and female consumers are represented by *B*_*f*_ and *B*_*m*_, respectively. The proportion of female consumers in the overall consumption of housing purchase in a family is referred to as θ, while non-housing expenditure is denoted to as *M*^2^. Furthermore, housing prices are implied by *p*_*R*_, and new housing demand by *R*.


(9) $$\begin{array}{l} o=\varphi P_{R}\bar{R} \end{array}$$

where *o* is the total value of existing houses, $\bar{R}$ is the number of existing houses, φ is the proportion of the total value of existing houses in total consumption, and φ can be 0. At this time, individuals tend to rent a house rather than buying a house. The budget is B, *B* + *c* = *B*_*f*_ + *B*_*m*_. An economy mainly seeks to maximize the utility of the whole (including men and women), and it needs to meet the following:


(10) $$\begin{array}{l} \Lambda =\text{λ}U_{f}+\left(1-\text{λ}\right)U_{m} \end{array}$$

Suppose the utility function of consumers is in the form of an invariant substitution elastic function:


(11) $$\begin{array}{l} U_{i}=\frac{{c_{i}}^{1-\varepsilon }}{1-\varepsilon },i=f,m \end{array}$$

For a family to maximize its consumption, it needs to meet the following conditions:


(12) $$\begin{array}{l} \underset{H,C}{\max}\Lambda \end{array}$$

(13) $$\begin{array}{l} s.t.P_{R}R+M=rk0+w+o \end{array}$$

Among them *r* is the interest rate of capital, and *k*0 is the capital endowment of a particular household. For the sake of convenience, it is presumed that there is no difference in the capital endowment of all households.

### Balance path analysis

Consumers ought to decide the direction of consumption according to the method of optimizing their utility. Suppose that *C* represents the time path of per capita consumption. Taking a representative consumer family with infinite survival as an example, the total utility TU obtained in its lifetime is as follows:


(14) $$\begin{array}{l} TU=\int_{0}^{\infty }e^{-\rho c}\left[\text{λ}U_{f}+\left(1-\text{λ}\right)U_{m}\right]dt \end{array}$$

The total output of the economy is *Y*, and its expression is *Y* = *K*^*α*^*L*^*β*^*R*^*γ*^. The constraint relationship between consumption, output, and capital is as follows:


(15) $$\begin{array}{l} \dot{K}=Y-C-R \end{array}$$

Let the new investment be *I*, $I=\dot{K}$, and *I* = *I*(*R, NR*). Where *R* denotes the value of land elements and *NR* represents non-land elements. It is assumed that the land in this study is mainly supplied for real estate construction. It means that *R* is proportional to *I*.

We assume the production function is Cobb–Douglas production function, that is, *Y* = *K*^*α*^*L*^*β*^. Taking the derivative of both sides of the function, we obtain the female and male wage rates:


(16) $$\begin{array}{l} w_{f}=\beta {K_{f}}^{\alpha }{L_{f}}^{\beta -1};w_{m}=\beta {K_{m}}^{\alpha }{L_{m}}^{\beta -1} \end{array}$$

In (16) 0 < *α* < 1, 0 < *β* < 1, assuming that there is an optimal wage level.

Then take the derivative of*L*_*f*_ and *L*_*m*_at both sides of (16), and then we constructed Hamilton's equation (details are given in the Appendix I section) to obtain the growth rate of consumption, which can be expressed as follows:


(17) $$\begin{array}{l} g_{c}=\frac{ċ}{c}=\frac{1}{\varepsilon }+\frac{\left(P_{R}-\varphi P_{R}\right)\beta {k_{f}}^{\alpha }{L_{f}}^{\beta -1}}{\varepsilon \left[\beta^{2}{k_{f}}^{\alpha }{L_{f}}^{\beta -1}-\zeta \left(L_{f}\right)\right]}-\frac{\rho }{\varepsilon } \end{array}$$

Given the linear relationship between consumption and output, the growth rate of consumption is equal to the economy's output growth rate, namely $g_{c}=\frac{\dot{c}}{c}=g_{Y}=\frac{\dot{Y}}{Y}$.

First of all, according to formula (16), if $\left(P_{R}-\varphi P_{R}\right)\beta {k_{f}}^{\alpha }{L_{f}}^{\beta -1}$ increase, *g*_*c*_ will also increase. Put it in another way, rising house prices will boost the economic growth rate, which leads to corollary 1.

This article mainly discusses the impact of the FLP rate on economic development and the role of housing prices in it. A detailed analysis of such propositions (corollaries 1–4), along with derivations, is given in equations A1–A12 in Appendix 1.

Corollary 1: Other things being equal, rising housing prices will promote economic's growth.

Corollary 2: Without taking into account other factors, an improvement in the FLP rate during the phase of economic and social development boosts economic development.

Corollary 3: Although FLP can promote economic development, the FLP rate appears to reduce the economy's driving force due to the restrictive effect of housing prices at a certain level.

Corollary 4: Under certain conditions, an increase in the FLP rate will promote economic development. The relationship between the FLP rate and economic growth is U-shaped; the higher the FLP rate, the less it can promote economic growth.

What should be emphasized is that in corollary 3, the improvement of the FLP rate can contribute to a supporting impact on economic growth. However, in conjunction with corollary 4, due to the inhibition of housing prices, FLP rates impede economic growth. We believe that housing prices can drive economic growth but will have negative effects on FLP in economic activities, and the inverted U-shaped relationship between the FLP rate and economic growth is primarily induced by housing prices.

### Data sources and descriptive statistics

This study uses data from the 2017 CFPSs, which were conducted by Peking University's China Social Science Survey Center (ISSS). The CFPS is a national household survey^3^ that represents Chinese society using multistage probability proportional to size (PPS) sampling with implicit stratification and rich socioeconomic data on individuals, families, and communities (Zhu et al., 2019). Because the focus of this study is on FLP, we considered females between the ages of 16 and 55, and these constraints resulted in a sample of 5,387^4^. The macro-data on housing prices are derived from the China Statistical Yearbook. This study used 1998 as the base year and processed housing price data to remove the effect of inflation using the Urban Residents Consumer Price Index (CPI). Table 1 shows summary statistics.

**Table 1:** Descriptive statistics of the variables.

**Variable**	**Obs**.	**Mean**	**Std. Dev**.	**Min**.	**Max**.
per capita GDP	5,387	55327.14	28089.82	27508	115613
Labor participation	5,378	0.7858	0.4103	0	1
House loof house ms	5,387	8.7602	0.4087	8.3680	10.2218
My father is still alive	5,387	0.5912	0.4917	0	1
The mother is still alive	5,387	0.9347	0.2472	0	1
Marriage status	5,387	0.6763	0.4916	0	1
Career category	5,387	1.4986	3.55741	1	5
Age	5,387	35.7462	8.861815	16	55
Health status	5,387	0.7256	5.8758	0	1
Number of children	5,387	1.3074	0.9510	0	7
Region	5,387	2.2645	1.0244	1	4

Source: China Family Tracking Survey 2017, China Social Science Survey Center, Peking University.

The main dependent variable in this study is the FLP, which is calculated using binary virtual variables with participation set to 1 and non-participation set to 0. Following Zhao (2013) and He (2015), we use the average sales price of commercial housing in the area where the interviewees' families live to measure housing prices. It can effectively prevent potential joint bias and alleviate housing prices and FLP in decision-making, as well as the cumulative causal effect of economic development. Housing prices are exogenous to individual households, avoiding potential joint bias and reducing the causal relationship between housing prices and FLP in decision-making and economic development. Furthermore, we follow Chen and Chen (2018) in using labor productivity as a proxy for economic development rather than actual per capita GDP. At the individual and household levels, we controlled for age, occupational category, marital status, health status, political affiliation, family structure characteristics, the number of children in the family, the presence or absence of living parents, and other individual characteristics. In addition, we considered whether they resided in the Eastern, Western, Central, or Northeastern regions.

This study assigns a weight of 0 to unmarried and alternative marital statuses, such as married, divorced, widowed, or separated, a weight of 1. In addition, health status is a binary variable. Individuals' health records are assigned a value of 0 if they have been unable to perform their usual activities for the last 4 weeks due to illness; otherwise, the value is 1. Following the occupation category, a value of 1 indicates self-sufficient agricultural production, a value of 2 indicates private enterprise, a value of 3 indicates individual enterprise, a value of 4 indicates employment, and a value of 5 indicates the number of children in non-farm scattered families, with a minimum of 0 and a maximum of 7, and an average of 1.30.

## Empirical analysis

We develop the following Probit discrete choice model to examine the net effect of housing price on FLP decision-making:


(1.a) $$\begin{array}{l} pr\left(FLFP-1\right)=\alpha +\beta \ln\;\left(hou\sin gprice\right)+\gamma X+U. \end{array}$$

The binary variable FLP in model (a) represents the status of FLP and has a value is 0 or 1. A value of 0 indicates that the female individual is not involved in labor; however, a value of 1 indicates the female has participated in labor. *α* refers to a constant term, represents the net effect of housing prices on the probability of FLP, and represents the housing price variables. Whereas *X* represents a set of control variables, including individual characteristics, family characteristics, and regional characteristics, such as whether it is located in the Eastern, Central, Western, and Northeastern regions, *U* is a random error term.

This study seeks to establish the following hypotheses: First, it will examine the effect of housing prices on FLP. Second, this study proposes an intermediary effect to determine whether FLP impacts economic development *via* housing price factors. Third, it will determine whether a threshold effect, defined as exceeding the value of a threshold, would mitigate the impact of FLP on economic development.

There is a potential endogeneity issue that could impact the study's findings. First, there could be a problem of reverse causality between rising housing prices and FLP. Because FLP and housing decisions are frequently made concurrently, rising housing prices may influence FLP decisions. For example, if housing prices are rising quickly, both husbands and wives may need to work to finance their housing improvement plans. At the same time, it is equally possible that an increase in labor supply results in homebuyers with a greater ability to make payments, which could increase housing prices. To address the aforementioned issue, we used an instrumental variable method. A valid instrumental should be highly correlated with rising house prices but uncorrelated with FLP decisions. To this end, this study used the average annual growth rate of housing prices over the previous 5 years as the housing prices instrument because growth rates in house purchasing prices could be closely related to future housing price appreciation. At the same time, the average annual growth rate of housing prices in the past should have been unrelated to FLP decisions made many years later, especially for households without a mortgage loan. This is especially true in urban China, where most people are hesitant to incur debt (Deng et al., 2008).

### Benchmark regression analysis

Table 2 shows the results of Probit estimation. We first examined the effect of housing prices on FLP at the national level. Then, by dividing the sample into four subregions—Eastern, Central, Western, and Northeastern regions—we investigate the relationship between housing prices and FLP at the regional level in order to determine how the cost of housing influences women's decision-making and whether heterogeneity exists. The reported results of the full-sample regression (column 1) show that the *ln* *hou*sin*gprice* has a significant positive impact on FLP, indicating that a unit increase in housing prices would increase the probability of FLP by 0.186%. These results are in line with the findings of Glaeser and Nathanson (2015). The underlying reason is that the purchase of a home and rising housing prices encourage households to reduce consumption while forcing households to increase labor supply (particularly FLP) and perform additional labor in order to increase labor income. However, our findings differ from those of Wu et al. (2016), who concluded that a 1% increase in housing prices would result in an average 0.08% decrease in FLP. Moreover, the empirical evidence suggests that housing prices have a positive effect on FLP in Eastern regions. That is, each unit increase in housing prices is associated with an average 0.22% increase in the probability of FLP.

**Table 2:** Results of the impact of housing price on FLP.

**Variables**	**Whole China** **(1)**	**Whole China** **(2)**	**Eastern region** **(1)**	**East** **(2)**	**Central** **(1)**	**Central** **(2)**	**West** **(1)**	**West** **(2)**	**Northeastern regions** **(1)**	**Northeastern regions** **(2)**
Lnhousingprice	0.186^***^ (3.91)	0.119^**^ (2.04)	^***^0.168 (3.19)	0.221^**^ (2.44)	−0.0001^*^ (−1.65)	−0.452 (−0.98)	−0.870^*^ (−1.52)	−0.220 (−0.32)	14.246 (1.58)	17.857 (1.06)
Father alive		−0.065^*^ (−1.73)		−0.127 (−1.45)		−0.0102 (−0.12)		−0.0592 (−0.63)		−0.0868 (−0.56)
Mother alive		−0.3647^***^ (−3.27)		−0.5916^**^ (−2.22)		−0.2947^*^ (−1.59)		−0.28568^*^ (−1.38)		−0.3355^*^ (−1.30)
Marriage		−0.06652 (−1.22)		−0.0616 (−0.65)		−0.0064 (−0.06)		−0.0802 (−0.73)		−0.33551 (−1.89)
Career classification		0.2144^***^ (27.25)		0.2443^***^ (14.87)		0.2060^*^ (15.57)		0.1936^***^ (12.09)		0.2328^***^ (10.32)
Party member or not		0.3061^***^ (2.59)		−0.00419 (−0.22)		0.64735^***^ (2.60)		0.23787^***^ (1.13)		0.4014 (1.00)
healthy state		0.0146^**^ (3.75)		0.0063 (0.95)		0.0135^**^ (1.82)		0.03117^***^ (4.02)		0.4014 (0.73)
No. of children		0.32683^***^ (10.99)		0.3583^***^ (6.47)		0.396^***^ (7.46)		0.237^***^ (4.35)		0.3993^***^ (3.21)
Age		0.0008 (0.38)		−0.0095^**^ (−2.50)		0.0023 (0.59)		0.0013 (0.32)		0.0044 (0.69)
Intersection	−0.860^**^ (−2.06)	−0.708 (−1.35)	−0.6904 (−1.9)	−1.291^*^ (−1.5)	−0.0001^***^ (3.09)	3.632 (0.91)	8.324^**^ (1.69)	2.6337 (0.45)		−152.8 (−1.06)
Province fixed	Yes	yes	Yes	Yes	Yes	Yes	Yes	Yes	Yes	Yes
Time fixed	Yes	yes	Yes	yes	Yes	Yes	Yes	Yes	Yes	Yes
LR	15.74	1,266.03	8.19	467.98	2.77	415.75	2.33	238.41		238.41
Pseudo R2	0.0029	0.2523	0.0035	0.2865	0.0022	0.2586	0.0022	0.2042	0.0009	0.3313
OBS	5,387	5,387	1,617	1,617	1,413	1,413	1,672	1,672	6,85	6,85

The marginal effect is reported in the table, and the *z*-statistic is in brackets.

***, **, and * are significant at the 1, 5, and 10% levels, respectively, and OBS is the number of samples. The other regression tables in this article are the same as this annotation.

Source: Calculated by the authors.

The national sample and the Eastern region sample both confirm the previous theoretical model inference: A rise in housing prices will promote FLP. It stems from a variety of factors, including the 1998 housing reform, which resulted in the expansion of the Chinese property market, as well as a number of public programs designed to expand the housing market and make affordable housing available to people of all income levels. In addition, employers were also encouraged to subsidize housing for their employees, as were real estate developers (Fu et al., 2016). The housing slave effect produced by the increase in housing prices prompted women from no-housing families to increase labor participation and supply. Thus, the empirical findings in Table 1 show additional support for theoretical hypothesis 2: FLP in Chinese cities is likely to increase as housing prices rise. The housing slave effect caused by rising housing prices encouraged women from low-income families to increase labor participation and supply. Thus, these empirical findings lend support to the theoretical hypothesis 2: FLP is likely to rise in Chinese cities as housing prices rise.

In contrast, the empirical evidence indicates that housing prices in the Central and Western regions have a negative but insignificant impact on FLP (columns 3 and 4). The underlying reason could be that housing costs in the Central and Western regions have risen too quickly, and women are not participating in labor in these regions. Perhaps, they would rather work in the east than locally. For instance, a person can earn money or remuneration by working in cities such as Beijing, Shanghai, and Guangzhou, and then purchase a house near the location of their household registration. Moreover, the housing prices have a positive but insignificant impact on FLP in the Northeastern region. It is possible that the samples in the Northeast are insufficient, necessitating additional research in the region.

The magnitudes and signs of the control variables are reasonable. For example, the coefficients of the father and mother alive variables are negative. Because of Chinese traditional culture, women will look after the elders in the family. This, in turn, reduced the probability of FLP. Furthermore, marriage status and age have no discernible effect on FLP, while health status and the number of children in the family both promote FLP and employment. Furthermore, the results reveal that the probability of FLP of party members is higher than that of non-party members. For example, in the national model (2), the coefficient of party members (0.3061) suggests that housing prices have a significant effect on FLP. The same holds true in three regions: Eastern, Central, and Western regions. This is because the party membership has a substantial positive effect on urban citizens' formal employment in state-owned units. Thus, the probability of party members' work in state-owned units is greater than that of non-party participants (Yan, 2017).

This study investigates the impact of housing costs on economic growth in more detail. Theoretical research revealed in this article suggests that housing prices have a moderate impact on economic growth. The coefficients of other control variables on economic development are not included to simplify the analysis. This shows that the effect of housing prices on economic development is being investigated solely without taking FLP into account. Table 3 shows the empirical outcomes. The results shown in Table 3 demonstrate that the *Lnhousingprice* coefficients are all positive and statistically significant at 1% (for all national or regional samples), indicating that housing prices have a positive impact on economic development. These findings are consistent with the findings of Fu et al. (2016). The results also indicate that the housing prices in the Northeast and the east have had the greatest impact on economic development, followed by the Western and Central regions. The study emphasizes that, if no other factors are taken into account, price increases alone will contribute to positive changes in regional economic development in a relatively short period of time. However, the perception that higher prices have greater positive effects on economic growth tends to diminish over time (Zhang, 2017).

**Table 3:** Results of the impact of housing price on short-term economic development.

**Variables**	**Whole China**	**East**	**Central**	**West**	**Northeast**
Lnhousingprice	5.883^***^ (1.215)	4.597^***^ (4.756)	2.431^***^ (1.215)	4.431^***^ (0.80)	4.727^***^ (1.414)
Constant	−4.601^***^ (−1.083)	−3.364^***^ (−0.376)	−1.664^***^ (−0.186)	−3.410^***^ (−0.791)	−4.0054^***^ (−1.397)
Province and time fixed effects	Yes	Yes	Yes	Yes	Yes
Pseudo R^2^	0.7328	0.5831	0.2759	0.0437	0.2252
OBS	5,387	1,617	1,413	1,672	685

Source: Calculated by the authors.

*** Stands for significant levels of 1%.

### Threshold effect analysis

This study further attempts to determine the effect of FLP on economic development, as well as whether FLP in economic activities is mediated by housing prices, and whether high housing prices alter the probability of FLP. The threshold measurement model is given as follows:


(1.b) $$\begin{array}{l} rgdp_{it}=c+FLFP+\beta_{1}x_{it}•I\left(\ln\;hou\sin gprice\leq \gamma \right)+\beta_{2}x_{it}\\ \;•I\left(\ln\;hou\sin gprice≻\gamma \right)+\varepsilon_{it} \end{array}$$

where *rgdp* indicates per capita GDP, which measures economic development; i and t indicate the region and time, respectively. Ln*housingprice* is the threshold variable in the threshold model. *γ* is the threshold value to be estimated. *β*_1_ and *β*_2_ signify the coefficients of the corresponding variables. *I*() is an indicative function, *ε*_*it*_ is a random variable, subject to independent and identical distribution.

The results of the threshold model are shown in Table 4. The entire threshold model has a *p-*value of 0, indicating that the model is highly reliable. The reported results show that the FLP coefficient is initially positive (5,047.087) and then becomes negative (−2,099.605), indicating that as urban housing prices rise, the FLP rate's contribution to economic development shifts from positive to negative. Thus, the threshold measurement results confirm the assertion of Tsani et al. (2013) that there is a U-shaped relationship between FLP and economic development. All variable *p-*values are zero, indicating that the obtained threshold is a one-layer threshold model with a threshold value of 8.813 for the housing prices logarithm. The underlying reason is that attracting and retaining a skilled workforce that will support economic growth requires the right housing offer (lower or affordable housing prices). The FLP's effects would increase the supply of labor, thereby reducing production costs by decreasing labor costs and wages. In addition, lower labor costs stimulate private consumption, which in turn stimulates private investment and drives GDP expansion. The influx of skilled labor can expedite economic transition and upgrading, leading to high-quality development. However, the flip side is that rising housing prices would decrease FLP, constrain labor supply, and reduce their spatial allocation efficiency. Either female workers would prefer to work in a location with lower housing costs, or they would leave the labor market. This, in turn, would cause a decline in economic growth.

**Table 4:** Threshold measurement results.

**Rgdp**	**Coef**	**Std.err**	**Z**	**P**	**Interval**
FLFP (1)	5,047.087	1,422.074	3.55	0.000	[2,259.873, 7,834.301]
Constant (1)	36,966.39	374.985	98.58	0.000	[36,231.43, 3,7701.35]
FLFP (2)	−2,099.605	421.442	−4.98	0.000	[−2,925.616, −1,273.594]
Constant (2)	84,841	1,252.137	67.76	0.000	[82,386.86, 87,295.14]

Source: Calculated by the authors.

Our empirical findings support the theoretical model's derivation that housing prices, as an intermediate medium, are crucial for women's economic participation. In addition, when jobs with inelastic or low elastic labor supply are excluded from the robustness test, it is discovered that the marginal effect of housing prices on the probability of FLP is also influenced by individual occupation characteristics. When housing prices fluctuate, FLP in decision-making may be extremely low or non-existent for civil servants, public institutions, and other occupations with a limited or even inelastic labor supply. The effect of housing prices on the probability of FLP remains stable after excluding the sample with low elastic or inelastic labor supply, the effect coefficient remains positive, and the effect has remained stable over time.

### Robust tests

We proceed to instrumental variable estimation to address the potential problem of endogeneity. Our housing prices instrument variable is the average annual growth rate of housing prices over the past 5 years (Table 5). In the first stage, the results reported in columns (1) and (3) indicate an increase in the average annual growth rate of housing prices. In the second stage, results are shown in columns (2) and (4); the housing price has a positive and statistically significant impact on FLP. Similar to the results of the baseline regression, these findings indicate that the effect of housing price on FLP is positive, statistically significant, and with a slightly different magnitude.

**Table 5:** Effect of housing prices on FLP.

	**Whole China**		**Eastern China**	
	**IV**		**IV**	
	**1^st^ stage**	**2^nd^ stage**	**1^st^ stage**	**2^nd^ stage**
lnhousingprice		0.105^***^ (0.020)		0.198^**^ (0.092)
lnAnnGrHP	0.742^***^ (0.049)		1.203^***^ (0.039)	
First stage F-stats	32.23		86.5	
R-squared	0.379	0.682	0.075	0.092
Control variables	Yes	Yes	Yes	Yes
Observations	5,387	5,387	1,617	1,617

*** Stands for significant levels of 1%.

We further use double-hurdle model^5^ to validate the effect of housing prices on the FLP rate, and the empirical results are shown in Table 6. The measurement model (1) employs a double-hurdle model with no control variables and shows that the housing prices promote the FLP rate at a 5% significance level. Model (2) indicates the effect of the housing prices with the control variable, and the coefficients of the housing prices are 0.162 and 0.186, respectively, indicating that the housing prices have a positive effect on FLP. Overall, our robustness tests confirm that housing prices have a significant positive effect on FLP in China.

**Table 6:** Double-hurdle model of housing prices affecting the FLP rate.

**Variable**	**(1)**	**(2)**
L n hp.	0.162^**^ (0.37)	0.186^**^ (0.45)
Control the variable	No	Yes
The wald value	110	116
Sample size	5,387	5,387

Source: Calculated by the authors.

** Stands for significant levels of 5%.

China has a lengthy history of financial autonomy. The majority of Chinese people regard housing as a “status” good and value homeownership. In the 1990s, housing privatization reforms led to unprecedented one-way increases in the real estate market, resulting in a substantial increase in the housing wealth of existing homeowners. Contrary to economic theory, however, rising housing prices have not resulted in a “wealth effect” that has decreased labor supply. Rather, the rapid increase in housing costs encouraged couples to become more involved in the labor market. Moreover, owning a home is one of the most important “status” items in China, which has a long tradition of valuing home ownership. For both urban and rural young people, homeownership is typically a prerequisite for marriage (Du, 2008; Zhang, 2016). The shifting demographics heightened competition in the marriage market. Given that housing is a significant cost associated with marriage and that marriage is one of the most important goals for young people, an increase in housing costs will encourage young people (especially females) to enter the labor force (Zhao et al., 2018). Furthermore, after a decade of consistent price growth, people now believe that buying a home is one of the most secure forms of investment. They consider the purchase of a home to be an investment with a relatively high rate of return when compared to other types of investments.

The rising cost of housing has an impact on the actual costs of businesses as well as the life quality of workers. It has an impact on urban labor concentration and dispersion, as well as the clustering and structure of local industries. Rising urban housing costs may hamper worker influx and reduce their spatial allocation efficiency. In general, cities with higher housing costs have better urban infrastructure and working environments. The polarization of social resource allocation will be made worse by the wealth effect of housing prices (Garretsen and Marlet, 2017).

## Conclusion

Since the 1990s housing privatization reforms, the Chinese housing market has experienced rapid price appreciation, resulting in a significant increase in the housing wealth of existing homeowners. However, rising housing prices did not result in a wealth effect, which reduces labor supply, but rather in an increase in labor supply, which contradicts the economic theory. In this study, we used a theoretical neoclassical economic development model that included housing consumption factors to estimate the effect of housing price dynamics on female labor participation (FLP), and we looked at the intrinsic relationship between gender and urban housing prices and economic development through the lens of the balanced growth path. For the empirical analysis, we used data from the 2017 CFPSs database (micro-database).

Our theoretical and empirical analysis yielded the following main results. The theoretical model demonstrated that, under certain conditions, an increase in the price of urban housing would increase the FLP rate, which in turn stimulates economic development. However, FLP and economic development have an inverted U-shaped relationship in China. Furthermore, the empirical analysis revealed that a unit increase in housing prices would result in a 0.186% increase in the Chinese FLP. Moreover, we documented that housing prices have a heterogeneous impact on economic development in the Eastern, Central, and Northeastern regions of China. Finally, the threshold model pointed out that FLP's economic development can be positively influenced up until the housing prices logarithm reaches 8.8134. However, the beneficial effects of FLP on economic development will be hampered once 8.8134 is reached. Therefore, we concluded that it is not always true to say that the higher housing prices are, the more economic development can occur.

Our findings have clear ramifications for the future development of the housing market and the strengthening of real estate market reforms, especially in Central, Western, and Northeast China, where it is necessary not only to increase the housing supply but also to stabilize employment. More importantly, priorities should be given to expanding employment opportunities, encouraging more women to work locally, and encouraging them to purchase houses in their local communities. In order to prevent a negative impact on FLP in economic activities, housing prices should not be allowed to rise too quickly, and there should be a limit. In addition, we should bring women's development into the framework of high-quality development, pay attention to women's education, improve the quality of women's human resources, and enhance women's labor productivity and their willingness to participate in labor. Moreover, we should speed up the construction of the aging care system and child care services, reduce the family burden and negative externalities faced by women in the process of participating in the labor force, and improve the external environment conducive to women's development. It is emphasized that policies concerning reasonable housing price planning should be developed in order to improve urban competitiveness and attract high-quality labor.

This study has certain limitations. One limitation of the current study is that the data used do not permit us to investigate the effects of rising housing prices on labor supply decisions for households with two or more housing units, a topic that merits further study when more detailed data become available. Given our findings, future research could look into the effect of rising housing prices on job selection and the duration of unemployment among homeowners.

## Data availability statement

The raw data supporting the conclusions of this article will be made available by the authors, without undue reservation.

## Ethics statement

Ethical review and approval was not required for the study on human participants in accordance with the local legislation and institutional requirements. Written informed consent from the patients/participants or patients/participants legal guardian/next of kin was not required to participate in this study in accordance with the national legislation and the institutional requirements.

## Author contributions

Conceptualization done by JK. Validation, formal analysis, investigation, resources, and data curation performed by JK and QM. Methodology, software, writing–original draft preparation, writing–review and editing, and policy recommendations done by YL and JK. All authors contributed to the article and approved the submitted version.

## Footnotes

^1^Asian Development Bank, Gender Equality and the Labor Market: Women, Work, and Migration in the People's Republic of China (2017).

^2^For the sake of simplicity of analysis, the price of non-housing consumption is not discussed in this study.

^3^Except for Hong Kong, Macao, and Taiwan, the sample is nationally representative, covers 31 provinces, municipalities, and autonomous regions, and can be combined with the macro-database to form a unified system.

^4^Keeping in mind that unrelated data are stored in the CFPS and assigned the value of −8 by the database designer is essential. For the purposes of analysis, irrelevant information may be omitted.

^5^The double-hurdle model is an extension of the Tobit model. As the model's name suggests, subjects must cross two hurdles to contribute. To be a potential contributor, the first hurdle must be overcome. The first (labor force participation) hurdle occurs when individuals decide whether or not to join the labor force. The second (employment) hurdle is when a person decides to work for a set wage or when employers select individuals from a queue.
