# Supplementary material for: Urban housing prices, female labor participation, and economic development in china: A theoretical and empirical analysis
Source: Front Psychol. 2023 Jan 4;13:970039. doi: 10.3389/fpsyg.2022.970039 (PMC9847388; doi:10.3389/fpsyg.2022.970039)
Supplement: Supplementary file 1 [file Presentation_1.pdf]

Supplementary material:

We take the derivative of L_f and L_m at both ends of (16), take 0, and set up the equation (2) in parallel to obtain information about males and females. Expression of labor participation rate relationship is as follow:

$$\frac{1+\upsilon l_m}{1+\upsilon l_f}=(\frac{k_m}{k_f})^{\alpha}(\frac{l_m}{l_f})^{\beta-2} \qquad \text{(A1)}$$

According to the analysis of (17), if $\frac{k_m}{k_f}$ is relatively constant, when human capital υ increases, $\frac{l_m}{l_f}$ will also increase. It also assumes that women's labor force rate would rise appropriately if women's human capital in society grows. That is, women are often likely to take part in economic and social activities.

Hamilton's equation can be constructed as followed:

$$\begin{aligned}&H=e^{-\rho t}(\Lambda(C_t))+\mu_A(Y+R-C)+\mu_B(1-t_0-t_f-L_f)+\\&\mu_D[P_R R+M-rk_0-w_f L_f-w_m(\frac{k_f}{k_m})^{\alpha}(\frac{L_f}{2-t-t_0-t_f})^{\beta-1}(1-t+t_f)-\varphi P_R R]\end{aligned} \qquad \text{(A2)}$$

μ_A, μ_B, μ_C and μ_D are "costate variables" describing the relationship between related variables. From the first-order condition: $\frac{\partial H}{\partial C}=0$; $\frac{\partial H}{\partial t_f}=0$; $\frac{\partial H}{\partial L_f}=0$ and Euler's equation $\frac{\partial H}{\partial R}=-\dot{\mu}_A$, we can get:

$$0=-\mu_A+e^{-\rho t}[\lambda(\theta C)^{-\varepsilon}+(1-\lambda)[(1-\theta)C]^{-\varepsilon}] \qquad \text{(A3)}$$

$$0=-\mu_B-[w_m(\frac{k_f}{k_m})^{\alpha}(\frac{L_f}{2-t-t_0-t_f})^{\beta-1}+(\beta-1)w_m(\frac{k_f}{k_m})^{\alpha}(\frac{L_f}{2-t-t_0-t_f})^{\beta-2}\frac{L_f}{(2-t-t_0-t_f)^2}]\mu_D \qquad \text{(A4)}$$

$$0=\beta K_f^{\ \alpha}L_f^{\ \beta-1}\mu_A-\mu_B-\beta^2 K_f^{\ \alpha}L_f^{\ \beta-1}\mu_D \qquad \text{(A5)}$$

$$-\dot{\mu}_A=\mu_A+(P_R-\varphi P_R)\mu_D \qquad \text{(A6)}$$

Among them, the proportion of female consumption in household consumption represent to as θ and $\lambda=\frac{L_f}{L_f+L_m}$ represents the female utility part amongst the overall utility. It is expected that the higher the FLP rate, the greater the λ is. After derivation, we can get:

$$\frac{-\dot{\mu}_A}{\mu_A}=1+\frac{(P_R-\varphi P_R)\beta k_f{}^{\alpha}L_f{}^{\beta-1}}{\beta^2 k_f{}^{\alpha}L_f{}^{\beta-1}-\zeta(L_f)} \tag{A7}$$

In (A7), $\zeta(L_f)=w_m(\frac{k_f}{k_m})^{\alpha}(\frac{L_f}{2-t-t_0-t_f})^{\beta-1}[1+(\beta-1)\frac{1-t+t_f}{L_f(2-t-t_0-t_f)}]$. Since $0<\beta<1$, $\zeta(L_f)$ is a decreasing function, then $\zeta(L_f)$ is greater than 0, $\frac{-\dot{\mu}_A}{\mu_A}=-\rho-\varepsilon\frac{\dot{c}}{c}$, namely $g_c=\frac{\dot{c}}{c}=\frac{-\dot{\mu}_A}{\varepsilon\mu_A}-\frac{\rho}{\varepsilon}$. Thus, we can get the growth rate of consumption, which can be expressed as:

$$g_c=\frac{\dot{c}}{c}=\frac{1}{\varepsilon}+\frac{(P_R-\varphi P_R)\beta k_f{}^{\alpha}L_f{}^{\beta-1}}{\varepsilon[\beta^2 k_f{}^{\alpha}L_f{}^{\beta-1}-\zeta(L_f)]}-\frac{\rho}{\varepsilon} \tag{A8}$$

This paper mainly discusses the impact of the FLP rate on economic development and the role of housing price in it. The following part will make a detailed analysis of such propositions. In the preceding part of the article, we have already assumed that $\zeta(L_f)=w_m(\frac{k_f}{k_m})^{\alpha}(\frac{L_f}{2-t-t_0-t_f})^{\beta-1}[1+(\beta-1)\frac{1-t+t_f}{L_f(2-t-t_0-t_f)}]$, if L_f increase, $\zeta(L_f)$ would decrease, leads to Corollary 2:

Corollary 2: In particular, without considering other considerations, an improvement in the FLP rate in the phase of economic and social development raises economic development.

In (A8), $g_c=\frac{\dot{c}}{c}=\frac{1}{\varepsilon}+\frac{(P_R-\varphi P_R)\beta k_f{}^{\alpha}L_f{}^{\beta-1}}{\varepsilon[\beta^2 k_f{}^{\alpha}L_f{}^{\beta-1}-\zeta(L_f)]}-\frac{\rho}{\varepsilon}$, When the denominator $\frac{(P_R-\varphi P_R)\beta k_f{}^{\alpha}L_f{}^{\beta-1}}{\beta^2 k_f{}^{\alpha}L_f{}^{\beta-1}-\zeta(L_f)}$ is 0, a critical value will appear. In other words, it is necessary to compare and judge $\beta^2 k_f{}^{\alpha}-w_m(\frac{k_f}{k_m})^{\alpha}(\frac{1}{2-t-t_0-t_f})^{\beta-1}\{1+(\beta-1)[\frac{1-t+t_f}{L_f(2-t-t_0-t_f)}]\}$ with 0. We take the derivation of L_f to the both sides of (A8) , then we get:

$$\frac{\partial(g_c)}{\partial(L_f)}=\frac{(\beta-1)(P_R-\varphi P_R)\beta k_f{}^{\alpha}L_f{}^{\beta-2}[\beta^2 k_f{}^{\alpha}-\zeta(L_f)]+\zeta'(L_f)(P_R-\varphi P_R)\beta k_f{}^{\alpha}L_f{}^{\beta-1}}{[\beta^2 k_f{}^{\alpha}L_f{}^{\beta-1}-\zeta(L_f)]^2} \tag{A9}$$

Because in (A9), ($\beta - 1$) is smaller than 0, $\zeta'(L_f)$ is also smaller than 0, So when P_R goes up, $\frac{\partial(g_c)}{\partial(L_f)}$ will go down. So the house prices will prevents the female labor force from pulling the economy forward, leads to corollary 3:

Corollary 3: At a certain stage, although FLP can foster economic development, the FLP rate appears to decrease the driving influence of the economy due to the restrictive effect of housing prices.

In corollary 2, We conclude that improvement in the women's labor force's participation rate may boost economic development, but the price has a specific inhibitory impact. Therefore, high housing prices do not promote economic development. Once the price of housing crosses a certain threshold, it will lead to a negative influence of women's participation to promote the economy. When g_c equal to zero, we can get the threshold value of the FLP rate. For the sake of simplicity of analysis, we change the form of $\zeta(L_f)$ according to the characteristics of itself, which can be expressed as follow: $\zeta(L_f) = AL_f^{\beta-1} + BL_f^{\beta-2}$, in it:

$$A = w_m(\frac{k_f}{k_m})^{\alpha}(\frac{1}{2-t-t_0-t_f})^{\beta-1} \qquad \text{(A10)}$$

$$B = (\beta-1)w_m(\frac{k_f}{k_m})^{\alpha}(\frac{1}{2-t-t_0-t_f})^{(\beta-1)}(\frac{1-t+t_f}{2-t-t_0-t_f}) \qquad \text{(A11)}$$

The economic growth rate of an economy can be defined as:

$$g_c = \frac{1}{\varepsilon} + \frac{(P_R - \varphi P_R)\beta k_f^{\alpha} L_f^{\beta-1}}{\varepsilon[L_f^{\beta-1}(\beta^2 k_f^{\alpha} - A) - B]} - \frac{\rho}{\varepsilon} \qquad \text{(A12)}$$

Thus we can get the critical value, when L_f is smaller than $\frac{[(\beta k_f^{\alpha}\frac{(P_R - \varphi P_R)\beta k_f^{\alpha}}{\rho - 1} + B]}{\beta^2 k_f^{\alpha} - A}$, g_c is greater than 0, when L_f is greater than $\frac{[(\beta k_f^{\alpha}\frac{(P_R - \varphi P_R)\beta k_f^{\alpha}}{\rho - 1} + B]}{\beta^2 k_f^{\alpha} - A}$, g_c is smaller than 0.

Therefore, we theoretically prove the inverted "U-shaped" relationship between female labor force participation rate and economic growth, and thus derive corollary 4:

Corollary 4: The improvement of FLP rate will promote economic development, but there is a certain

prerequisite for its driving role. The relationship between FLP rate and economic growth is U-type, higher the FLP rate is not the more it can promote economic growth.

What should be emphasized is that in corollary 3, the improvement of women's Labor participation rate can contribute to a supporting impact on economic growth. However, in conjunction with corollary 4, due to the inhibition of house prices, women's labor participation rates decrease a positive impact on economic growth. We believe that housing prices can drive economic growth but will have negative effects on women's participation in economic activities, and the inverted U-shaped relationship between FLP rate and economic growth is primarily induced by house prices.
